# Reliability of nociceptive monitors vs. standard practice during general anesthesia: a prospective observational study

**DOI:** 10.1186/s12871-025-02923-4

**Published:** 2025-01-31

**Authors:** Daniel Widarsson Norbeck, Sophie Lindgren, Axel Wolf, Pether Jildenstål

**Affiliations:** 1 Institute of Health and Care Sciences, Sahlgrenska Academy, University of Gothenburg, Gothenburg, Sweden; 2 Department of Anaesthesiology and Intensive Care, Sahlgrenska University Hospital, Gothenburg, Sweden; 3 Department of Anaesthesiology and Intensive Care Medicine, Sahlgrenska University Hospital/Östra, Gothenburg, Sweden; 4 Institute of Nursing and Health Promotion, Oslo Metropolitan University, Oslo, Norway; 5 Department of Hybride and Interventional Procedures, Sahlgrenska University Hospital, Gothenburg, Sweden; 6 Department of Health Sciences, Lund University, Lund, Sweden; 7 Department of Anesthesiology and Intensive Care, Örebro University Hospital and School of Medical Sciences, Örebro University, Örebro, Sweden

**Keywords:** Nociception monitoring, Minimally invasive abdominal interventions, Analgesia, Surgical pain management, Anesthesia monitoring, Nociception level index (NOL), Skin conductance algesimeter (SCA), PainSensor, Post-operative pain, And intraoperative analgesia

## Abstract

**Background:**

Inadequate or excessive nociceptive control during general anesthesia can result in significant adverse outcomes. Using traditional clinical variables, such as heart rate, systolic blood pressure, and respiratory rate, to assess and manage nociceptive responses is often insufficient and could lead to overtreatment with both anesthetics and opioids. This study evaluated the feasibility and effectiveness of three nociception monitoring techniques Nociception Level Index (NOL), Skin Conductance Algesimeter (SCA) and heart rate monitoring in patients undergoing image-guided, minimally invasive abdominal interventions under general anesthesia.

**Method:**

This prospective observational study collected data from 2022 to 2024. All patients were anesthetized according to the department’s routine, and predetermined events were recorded. Two commercially available nociception monitors, the PMD-200 from Medasense (NOL) and PainSensor from MedStorm (SCA), were used, and their data were collected along with various hemodynamic parameters. The three nociception monitoring techniques were compared during predetermined events.

**Result:**

A total of 49 patients were included in this study. NOL and SCA demonstrated higher responsiveness than HR for all events except for skin incision. The comparison of the values above and below the threshold for each nociceptive stimulus showed significance for all measurements using the SCA and NOL. However, using HR as a surrogate for nociception with a threshold of a 10% increase from baseline, the difference was significant only at skin incision. There was no variation in the peak values attributable to differences in patients’ age. Weight was a significant predictor of the peak NOL values.

**Conclusion:**

NOL and SCA demonstrated superior sensitivity and responsiveness to nociceptive stimuli compared to HR, effectively detecting significant changes in nociceptive thresholds across various stimuli, although responses during skin incision showed no such advantage.

**Trial registration:**

Clinical trial - NCT05218551.

**Supplementary Information:**

The online version contains supplementary material available at 10.1186/s12871-025-02923-4.

## Background

Evidence links general anesthesia to post-operative cognitive dysfunction (POCD) [1, 2], with nociception and anesthetic depth being key factors. Analgesics, especially opioids, mitigate nociception but their intra- and post-operative effects are not fully understood. Reliable nociceptive monitoring during surgery remains elusive, highlighting the need for better tools to tailor analgesia [3].

Inadequate or excessive nociceptive control can lead to adverse outcomes, such as Remifentanil-induced hyperalgesia and prolonged post-operative pain [4–6]. Individual variability in opioid response, influenced by genetics, underscores the need for personalized approaches [7–9]. With Europe’s elderly population expected to double by 2050 [1], refined anesthesia practices are critical to ensure safety and efficacy for this vulnerable group.

Heart rate is commonly used to assess nociception under anesthesia, but it is influenced by non-nociceptive factors, making subjective interpretation necessary [10–12]. Over-reliance on clinical parameters like heart rate, blood pressure, and respiratory rate may lead to overtreatment with anesthetics or opioids, affecting hemodynamic stability and complicating management [12–14].

Recent nociception monitors, such as the Medasense PMD-200 and MedStorm PainSensor utilizes parameters such as heart rate and/or skin conductance to objectively quantify nociceptive responses [15]. The PMD-200 provides a Nociception Level Index (NOL) ranging from 0 to 100, with 25 as the treatment threshold [16]. In contrast, the PainSensor, exemplified by the Skin Conductance Algesimeter (SCA) index, employs either a treatment threshold of 0.06 peaks/second or a scale ranging from 0 to 10, with a threshold of ≥ 3. These devices show potential but require further validation, particularly in complex patient populations [17, 18].

This study assessed the feasibility and effectiveness of three nociception-monitoring techniques—NOL, SCA, and heart rate monitoring—in patients undergoing image-guided minimally invasive abdominal interventions under general anesthesia.

## Aim

To evaluate the effectiveness of different somatic nociceptive monitoring techniques and visualize their responses to various nociceptive stimuli during minimally invasive procedures under general anesthesia.

### Patients and anesthesia methods

This was an observational study that included 49 patients. The assumption for the number of participants included in this observational study is based on calculations derived from the articles by Rantanen et al. [19] and Martini et al. [20].

Patients were screened for eligibility via medical records and asked to participate during the preoperative assessment visit at the hospital. All patients received written and oral information. Written informed consent was obtained from all participants. The trial conduct adhered to the Declaration of Helsinki and Good Clinical Practice guidelines.

The inclusion criteria specified adults aged 18 and older who had undergone minimally invasive abdominal interventions under general anesthesia with a small incision either in the abdomen or through the femoral artery or vein. Patients were excluded if they had an American Society of Anesthesiologists (ASA) risk classification > IV, chronic pain, or known neurological disorders [see Additional file 1].

All patients were anesthetized following the department’s routine, e.g., induction, Propofol^®^ 1–3 mg/kg IV and Remifentanil^®^ administered via a target-controlled infusion pump (Minto model) [21] with an effect site target (Ce) of 5.0 ng/ml IV, and Rocuronium^®^ 0.6 mg/kg IV were used. During the skin incision, the Ce of Remifentanil was set to 4.0 ng/ml. Anesthesia was maintained using an infusion of Sevoflurane and Remifentanil.

All patients received vasopressors via IV infusion with Norepinephrine 0.04 mg/ml and a crystalloid solution according to the department routine. Philips^®^ Intellivue X3 was used for measuring vital parameters, including pulse oximetry, invasive or non-invasive blood pressure, 3-lead ECG, NMT, and SedLine (4-channel processed EEG Masimo^®^ technology). Nociception was monitored before anesthesia induction. The MedStorm^®^ PainSensor was positioned on the patient’s left hand, and the Medasense^®^ PMD-200 (NOL) sensor was placed on the right or left hand.

### Data collection and procedure

All the data were collected at a university hospital in Sweden between 2022 and 2024. All parameters, drugs, and predetermined events (jaw-thrust, laryngoscopy, endotracheal tube insertion, and skin incision) were recorded. The minimal alveolar concentration of sevoflurane (MAC), end-tidal carbon dioxide concentration (EtCO_2_), and non-invasive blood pressure measurements (SBP, DBP, and MAP) were recorded every 5 min. The NOL Index was recorded every 5 s, and the depth of anesthesia was recorded every 2 s as spectral edge frequency (SEF-L and SEF-R), indicating that 95% of the EEG frequency was below the given Hz [22]. The SCA peaks/sec, pulse oximetry (S_p_O_2_), heart rate (HR), and invasive blood pressure (SBP, DBP, and MAP) were recorded every second.

The thresholds for commercial nociception monitors were based on manufacturer-specified values as the limits for initiating nociception treatment. The 10% increase in heart rate that we used for comparison with traditional clinical heart rate assessment via the monitor is not an established research-based limit but rather derived from clinical experience. However, a pilot study investigating similar stimuli showed that a 90% increase in NOL corresponded to a 12% increase in heart rate [23].

Hemodynamic and respiratory data were recorded using the Moberg CNS-200. Four devices (Medasense PMD-200, MedStorm PainSensor, Masimo SedLine, and Moberg CNS-200) were employed for data recording, and time synchronization among the devices was performed manually before each patient. All the recorded data were merged using a Python script in a Jupyter Notebook. The baseline to peak data (Table 2) were defined as the peak values recorded by the different monitors within ± 15 s of the event, occurring within a ± 30-second window from the registered event time, to minimize potential errors related to manual event marking and time synchronization between devices.

Data collection began with patient preoxygenation and ended when the patient was transferred after extubation. Nociceptive monitors were blinded to all staff throughout the data collection process.

### Calculation and statistics

The statistical methods utilized in this study included repeated measures ANOVA to assess changes over time, independent-samples t-tests to compare group means, and Pearson’s correlation coefficient to evaluate the relationship between continuous variables. A Receiver Operating Characteristic (ROC) curve analysis was performed to evaluate the different monitoring techniques and to calculate the Area Under the Curve (AUC). All statistical calculations, analyses, and visualizations were performed using IBM SPSS Statistics for MacOS (Version 29.0.2.0, IBM Corp., Armonk, NY, USA) and RStudio (Version 2024.04.2 + 764, RStudio, PBC, Boston, MA, USA). Descriptive statistics and basic calculations were performed using Jupyter Notebook 6.4.8 (Python 3.0) and Microsoft Excel (version 16.85).

## Result

All patients received the same level of analgesia based on department routines and variables such as weight, age, sex and height (Table 1).

The difference between the highest and baseline values was significant for all stimuli with all three measurement methods, except for SCA and HR during skin incision. However, NOL and SCA showed higher responsiveness than HR to all stimuli except skin incision (Table 2).

**Table 1:** Demographcis

Demographic	Number (*n*=)	Mean, (min–max):
Number of patients (n=):	49	-
Gender (m/f, n=):	31/18	-
Age, years (mean, min–max):	-	64 (31–87)
Weight, kg (mean, min–max):	-	81 (50–139)
Height, cm (mean, min–max):	-	174 (152–200)
Asa (i/ii/iii/iv, n=):	2/13/33/1	-
Use of adrenergic blockers and/or antihypertensive drugs	49	
Percutaneous transhepatic cholangiogram (ptc):	13	-
Transarterial chemoembolization (tace):	10	-
Hepatic artery embolization:	14	-
Other minimal invasive surgery:	12	-

**Table 2:** Median change from baseline to peak as per monitor and stimuli. The baseline was measured at a point of steady-state anesthesia (two minutes before the skin incision)

	NOL (index)	SCA (peaks/sec)	HR (beats/min)
Jaw-thrust *p-value*	16.00 (14.25) *< 0.001*	0.067 (0.165) *< 0.001*	12.00 (12.28) *< 0.001*
Laryngoscopy *p-value*	18.00 (18.63) *< 0.001*	0.067 (0.126) *< 0.001*	9.00 (11.38) *< 0.001*
Insertion of endotracheal tube *p-value*	20.00 (18.2) *< 0.001*	0.067 (0.151) *< 0.001*	10.00 (11.479) *< 0.001*
Skin incision *p-value*	2.00 (10.21) *< 0.001*	0.000 (0.077) *0.208*	1.00 (9.39) *0.106*

Comparison of the values above and below the threshold for each nociceptive stimulus showed significance for all measurements using the SCA and NOL. Hence, using HR as a surrogate for nociception with a threshold of a 10% increase from baseline, the difference was significant only at skin incision (Table 3).

**Table 3:** Median values ​​when reacting to stimuli (S1 = jaw-thrust, S2 = laryngoscopy and insertion of endotracheal tube, and S3 = skin incision). Pre/post values ​​are defined as the lowest value within 30 s before and after stimuli

	NOL	SCA	HR 10%*
S1 Pre	14,00 (10,45)	0,000 (0,062)	68,50 (13,73)
S1 Reaction	20,00 (12,16)	0,067 (0,147)	73,50 (13,35)
S1 Post	14,00 (10,63)	0,000 (0,025)	68,00 (13,11)
P (Repeated measures ANOVA)	< 0,001	< 0,001	< 0,001
S1 number over threshold	18**	21**	9
S2 Pre	16,00 (16,89)	0,000 (0,211)	64,00 (14,75)
S2 Reaction	24,00 (17,21)	0,067 (0,142)	71,00 (14,80)
S2 Post	21,00 (16,41)	0,000 (0,070)	67,00 (15,17)
P (Repeated measures ANOVA)	< 0,001	< 0,001	< 0,001
S2 number over threshold	23**	21**	7
S3 Pre	3,00 (723)	0,000 (0,367)	58,00 (11,07)
S3 Reaction	8,50 (11,74)	0,000 (0,079)	60,50 (13,33)
S3 Post	4,50 (10,13)	0,000 (0,020)	59,00 (10,78)
P (Repeated measures ANOVA)	< 0,001	0,009	0,003
S3 number over threshold	7**	4**	7

* Heart rate threshold 10% increase from baseline

** Threshold NOL > 25 and SCA > 0.06 peaks/sec

Between the two nociception monitoring techniques, the SCA recorded more patients above the threshold during jaw-thrust and fewer during endotracheal tube insertion (Table 3). For skin incisions, both nociception monitors showed a significantly lower number of patients above the threshold. However, NOL showed 57% more patients above the threshold than SCA. During jaw-thrust and endotracheal tube insertion, the SCA showed the same number of patients above the threshold for both stimuli, while the HR and NOL varied. Regardless of the stimuli, heart rate detected a consistent number of patients over the threshold (Table 3).

A Pearson correlation was made for all stimuli combined and showed a significant relationship between peak values for NOL and HR (p = < 0.001). A correlation analysis showed a strong and significant relationship for both NOL and SCA compared with HR (p = < 0.001 versus *p* = .002). When comparing the correlation between HR and NOL/SCA in peak values, the strongest predictor of elevated HR was an increase in SCA, although this was not significant (B = 7.48, *p* = .377). NOL peak values significantly predicted HR increase but had a lower beta value than SCA (B = 0.16, *p* = .025).

There were no significant differences in the peak values of NOL (*p* = .216), SCA (*p* = .313), or HR (*p* = .458) based on sex. However, there was a significant difference in the increase from baseline to peak with NOL at the group level (*p* = .015). There was no variation in peak values explained by age differences between patients in NOL (B = − 0.099, *p* = .254), SCA (B = 0.001, *p* = .286), or HR (B = − 0.053, p = < 0.001). Hence, weight was a significant predictor of peak values in the NOL technique used during nociceptive stimuli (B = 0.21, p = < 0.001).

**Fig. 1:**
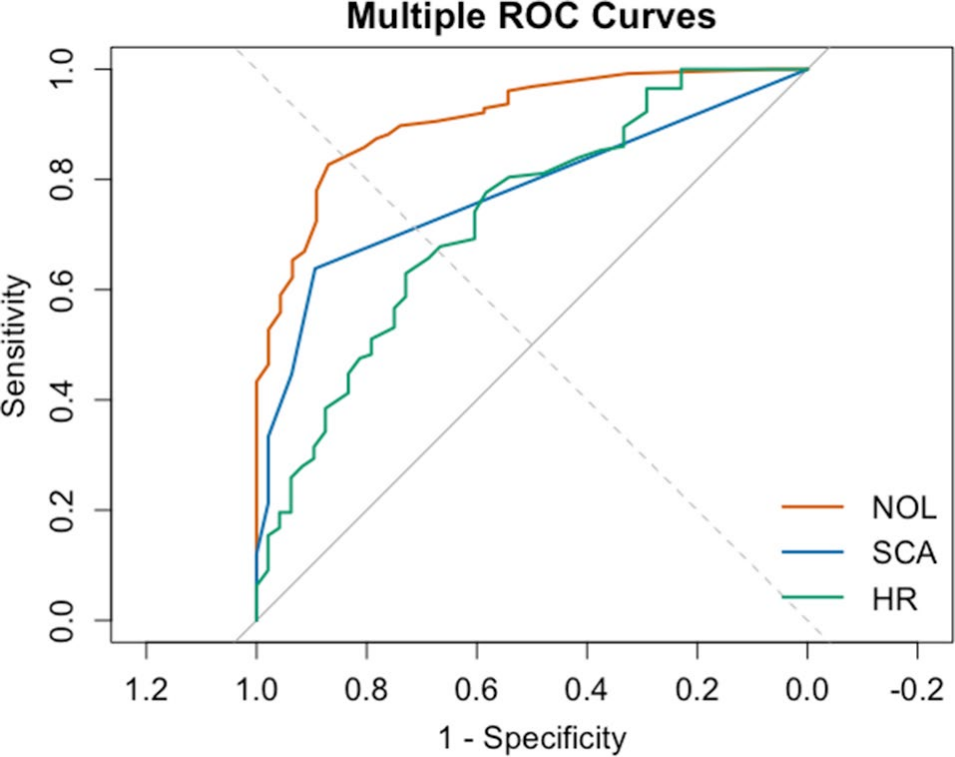
Showing receiver operating characteristics (ROC) curve of NOL, SCA and HR. ROC curve discrimination between nociceptive (jaw-thrust and intubation) and nonnociceptive stimuli at Remifentanil concentration (Ce 5/3 ng/ml). Sensitivity and specificity provided in Table 4

The ROC curves for NOL, SCA and HR (all measured after stimulation) were calculated (*n* = 168) and are displayed in (Fig. 1). Sensitivity values at a specificity of 75%, along with the areas under the ROC curves, are provided in (Table 4). Among the variables, NOL demonstrated superior performance in distinguishing between noxious (jaw-thrust and intubation) and non-noxious events, achieving an AUC of 0.89 (95% CI, 0.86-0.96) (Table 4).

**Table 4:** AUC, Sensitivity, PPV and NPV of the NOL and HR at a specificity of 75%

	AUC (95% CI)	Sensitivity (%)	Specificity (%)	PPV (%)	NPV (%)
HR	0.73 (0.65–0.81)	57	75	87	37
NOL	0.91 (0.86–0.96)	89	75	91	71
SCA	0.78 (0.72-0.083)	70	75	89	45

AUC = Area Under the Curve, PPV = Positive Predictive Value, NPV = Negative Predictive Value, HR = Heart Rate, NOL = Nociception Level Index, SCA = Skin Conductance Algesimeter

## Discussion

This observational study compared three somatic methods of assessing nociception in response to known tissue stimuli. These stimuli were jaw-thrust, laryngoscopy, insertion of the endotracheal tube, and skin incision, which are known to trigger stress reactions and are perceived as nociceptive and have been used in previous research [20, 24, 25]. Our study used the same relative doses of analgesics and the same types of stimuli across individuals with varying medical histories. The three methods observed, which serve as surrogates for nociception, generally responded similarly yet exhibited some significant differences. Our results indicate and supports by other studies [12, 16, 26–28] that the heart rate is not superior and, in some cases, is even less effective than commercial nociception monitors.

Accordingly, all our patients were prescribed various medications to manage blood pressure and heart disease (Table 1). While research [29] suggests that adrenergic blockers may have a more pronounced effect on the nociceptive response to moderate knee pain compared to other antihypertensive drugs, this claim is contradicted by Zhou et al., [30] who found no evidence that beta blockers provide a clinically meaningful reduction in knee pain. In our case, the impact of these different medications on patients with cardiovascular conditions under general anesthesia, particularly concerning their nociceptive response, remains unclear.

This study also showed that monitoring nociception has advantages over the heart for assessing nociceptive stimuli. The nociceptive-extracted values were two to three times as many patients above the threshold compared to heart rate, indicating a stronger correlation with the stimulus in relation to heart rate only. This finding is supported by a study showing that SCA is superior to changes in HR and blood pressure in critically ill mechanically ventilated patients [31].

Both the NOL and SCA have been validated for their response to tissue stimuli and different levels of analgesia [16, 32]. Studies [17, 33] have shown that NOL-guided analgesia can reduce intraoperative opioid consumption and positively impact the experience of post-operative pain. However, a systematic meta-analysis [34] has also been conducted, which failed to demonstrate a definitive benefit of NOL in improving patient outcomes, highlighting the necessity for further research to validate its efficacy across varied clinical contexts. In this study, the SCA showed great similarity to the NOL, though significant differences were observed between the devices, with the SCA showing more consistent results and independence from patient demographics.

Studies have shown that individuals with obesity exhibit altered nociceptive processing, potentially due to ongoing and heightened inflammatory responses compared to individuals with normal weight [35–38]. Our study suggests that obesity, as a risk factor, may influence nociceptive mechanisms and serve as a predictor for elevated NOL values. This finding may be attributed to the NOL technique, which appears to demonstrate higher sensitivity and specificity compared to SCA and HR measurements. Furthermore, our results align with the findings of the review article by Martinez-Vazquez P et al. [12].

To some extent, all patients in this study showed an increase in somatic stress, for example, nociception levels, when exposed to stimuli, and half exceeded the threshold for treatment, as described by the respective manufacturer (NOL and SCA). All three nociception assessment methods used in this study identified these increases to varying degrees. During stimuli with higher doses of remifentanil and longer duration (jaw-thrust, laryngoscopy, and insertion of an endotracheal tube), HR was inferior to nociception monitors. For the skin incision, which was a shorter stimulus, all three methods behaved similarly. This could be because the tissue stimuli were not sufficiently intense to trigger a nociceptive response [39].

Interestingly, not all patients experienced adequate nociceptive relief despite the administration of high doses of remifentanil [6]. Approximately half of the patients exhibited nociceptive values indicative of the need for treatment, suggesting that these individuals were subjected to levels of surgical stress that could potentially have adverse effects on post-operative recovery and well-being [40]. This, in combination with the risks of overtreatment with opioids, shows the importance of closely monitoring these complex patients, as relying solely on HR does not seem to capture the variations in nociception level with the same veracity as NOL and SCA. With older age, more comorbidities, and polypharmacy, the influence on hemodynamic events grows, making it more difficult to correlate with the state of the nociceptive response. As individuals age, the prevalence of comorbidities and the complexity of polypharmacy increase, which heightens the impact on hemodynamic events [41]. This interplay complicates the ability to accurately correlate hemodynamic changes with nociceptive responses.

During anesthesia administered in our department, norepinephrine is used as the first-line vasopressor, consistent with recommendations highlighted in the literature [42]. However, the algorithm for the conductance technique was not influenced by calcium levels, as the method employed for assessing skin sympathetic nerve activity (SSNA) is inherently independent of calcium-mediated effects [43].

Anesthesia personnel tend to prioritize hemodynamic stability over analgesia when treating patients under general anesthesia. For instance, Carella M et al. [44] emphasized the importance of maintaining hemodynamic stability during anesthesia, noting that it often becomes the primary focus for anesthesiologists. The authors stated, “We focused on the final systemic aspect of nociceptive stress response and considered hemodynamic stability to be our real challenge”. While ensuring nociceptive balance is essential, immediate management of hemodynamic parameters often takes precedence, highlighting the complexity of using HR as a measure of nociception [45].

Additionally, research has highlighted the challenges in using HR as an indicator of nociception. A study by Meijer et al. [46] discussed the use of nociception monitors to guide opioid administration, aiming to optimize anesthesia and improve patient outcomes. The study found that while these monitors can aid in assessing nociception, relying solely on HR can be problematic due to its sensitivity to various factors beyond pain, such as physiological and pharmacological influences.

This study demonstrated the utility of nociceptive measurements, which may enable the identification of individuals with genetic variations related to increased tolerance or heightened sensitivity to analgesic medications with greater precision regarding their nociceptive responses [7]. Consequently, these individuals can be managed individually, ensuring the administration of an appropriate pain regimen with higher accuracy.

We have studied the sensitivity and effectiveness of various methods for describing nociceptive responses during general anesthesia. Our study includes a ROC analysis for different stimuli, incorporating various methods such as NOL, SCA and HR. The results suggest that composite methods perform well, and even better than HR alone, in detecting nociception. However, the aim of this study is to describe clinically available and effective method/s for nociception detection during general anesthesia that have already been validated for sensitivity and specificity.

Our findings, along with those of other studies [17, 47], underscore the challenges of achieving effective analgesia during general anesthesia and the shortcomings of relying solely on heart rate as an indicator of nociception.

## Conclusions

This study examines the comparative effectiveness of three nociception monitoring methods—NOL, SCA, and HR—during standardized analgesia. NOL and SCA demonstrated superior sensitivity and responsiveness to nociceptive stimuli compared to HR, with the exception of responses during skin incision. Both NOL and SCA effectively detected significant changes in nociceptive thresholds across various stimuli, whereas HR exhibited limited variability and significance.

The findings indicate that NOL and SCA are superior to HR in detecting nociceptive changes, with NOL providing additional predictive value related to patient weight. These results are significant as they offer valuable insights for clinicians, enabling the selection of more reliable nociception monitoring techniques to enhance patient care during procedures requiring analgesia.

### Limitations

We recognized that there were different personnel performing the anesthesia. However, everyone followed the departmental routine, and no deviations that could affect the results were noted. We included a variety of minimally invasive abdominal interventions, but all patients included in the study received the same type of stimulus after anesthesia induction, except for the PTC group, which underwent a tissue incision in the abdomen instead of the groin. However, their nociceptive responses did not differ from those of the other groups and did not influence the overall results.

## Electronic supplementary material

Below is the link to the electronic supplementary material.

Supplementary Material 1: Additional file 1 - Inclusion chart. Figure 1 – ROC curve

## Data Availability

Data is provided within the manuscript or supplementary information files.

## Abbreviations

NOL: Nociception Level Index
SCA: Skin Conductance Algesimeter
HR: Heart Rate
ASA: American Society of Anesthesiologists physical status classification system
MAC: Minimal Alveolar Concentration
EtCO2: End-Tidal Carbon Dioxide
SBP: Systolic Blood Pressure
DBP: Diastolic Blood Pressure
MAP: Mean Arterial Pressure
SEF: Spectral Edge Frequency
SpO2: Oxygen Saturation
PRD: Pupillary Reflex Dilation
PTC: Percutaneous Transhepatic Cholangiogram
TACE: Transarterial Chemoembolization
