# Supplementary figures and images for: Reliability of nociceptive monitors vs. standard practice during general anesthesia: a prospective observational study
Source: BMC Anesthesiol. 2025 Jan 31;25:51. doi: 10.1186/s12871-025-02923-4 (PMC11783742; doi:10.1186/s12871-025-02923-4)

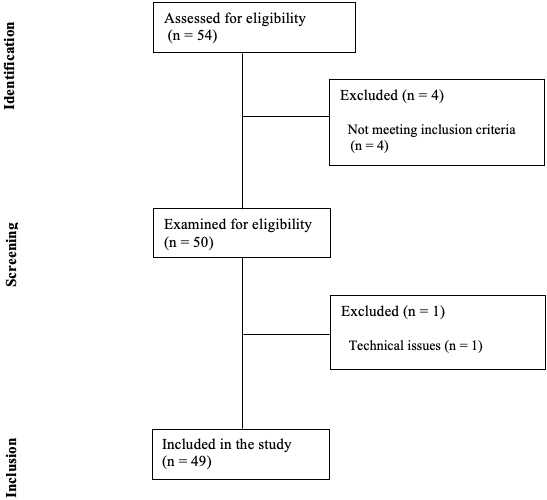

Supplement: Supplementary file 1 — Supplementary Material 1: Additional file 1 - Inclusion chart. Figure 1 – ROC curve [file 12871_2025_2923_MOESM1_ESM.png]
